# Association between non-high-density lipoprotein cholesterol to high-density lipoprotein cholesterol ratio and sarcopenia with mediating role of triglyceride glucose-related indices: a cohort study in Chinese elderly adults

**DOI:** 10.3389/fnut.2026.1876093

**Published:** 2026-08-05

**Authors:** Ping Zhang, Mengmeng Zheng, Yue Wang, Xiaoyu Pan

**Affiliations:** Department of Endocrinology, The Second Affiliated Hospital of Anhui Medical University, Hefei, Anhui, China

**Keywords:** cross-sectional study, elderly adults, NHHR, prospective cohort study, sarcopenia

## Abstract

**Background:**

The present study aims to investigate the association and dose-response relationship between the non-high-density lipoprotein cholesterol to high-density lipoprotein cholesterol ratio (NHHR) and sarcopenia in elderly adults. The ultimate objective is to thereby clarify its predictive value for sarcopenia risk.

**Methods:**

A cross-sectional study was conducted using data from the cohort of 368 elderly individuals aged 60 years and over, as part of the Second Affiliated Hospital of Anhui Medical University. A prospective cohort study was also conducted using CHARLS data, encompassing 3,152 elderly individuals aged 60 years and over, with no instances of sarcopenia at the initial baseline. The median follow-up period for this study is 4.5 years. The association was analyzed using both logistic regression and Cox proportional hazards regression, with the results of this analysis subsequently validated by restricted cubic spline (RCS) analysis and subgroup analysis. Subsequently, the mediating role of the triglyceride glucose (TyG)-related indices was elucidated through mediation analysis.

**Results:**

Following the adjustment of confounding factors, a unit increase in NHHR was observed to be associated with a 31% reduction in sarcopenia risk in cross-sectional studies (OR = 0.69, 95% CI: 0.56–0.86, P < 0.001). In prospective cohort studies, the risk of sarcopenia decreased by 37% (HR = 0.63, 95% CI: 0.51–0.77, *P* < 0.001). A stable linear inverse relationship was observed between the two variables, with no threshold effect, and subgroup analyses validated the robustness of this association. Additionally, the TyG-related indices exerted a significant mediating effect on the association between NHHR and sarcopenia risk, with a mediation proportion ranging from 22.48 to 29.47%.

**Conclusion:**

NHHR is significantly negatively correlated with the risk of sarcopenia in Chinese elderly adults and may serve as a potential biomarker for sarcopenia risk stratification. TyG-related indicators play a partial mediating role in this association.

## Introduction

The accelerating process of global population aging has resulted in a growing societal focus on health concerns among the elderly. Sarcopenia, a prototype geriatric syndrome, has become a major public health concern, exerting a detrimental influence on quality of life and escalating the burden of healthcare. This progressive systemic disorder has its origins in a number of factors, including the process of aging, inadequate nutritional intake, reduced physical activity, and the presence of chronic diseases. Its core characteristics comprise diminished skeletal muscle mass, decreased muscular strength, and/or impaired physical function (1). A global prevalence of sarcopenia among individuals aged ≥ 60 years has been indicated by epidemiological data, ranging from 10 to 20%, with a marked upward trend with increasing age. Amongst the very elderly aged ≥ 80 years, the prevalence of sarcopenia has been reported to exceed 30% (2). Sarcopenia, a condition characterized by the loss of muscle mass and strength, has been shown to result in a range of adverse outcomes for older adults. These include muscle wasting, weakness, and diminished mobility. Furthermore, sarcopenia has been demonstrated to substantially increase the risk of falls, fractures, disability, hospitalization, and even all-cause mortality. Research indicates that individuals with sarcopenia face a two to threefold higher mortality risk compared to those without the condition (3), imposing a significant burden on personal health, family care, and healthcare systems. Therefore, the identification of risk factors for sarcopenia, the exploration of biomarkers capable of early prediction of sarcopenia risk, and the development of scientifically sound and effective early prevention and intervention strategies hold significant clinical and public health value for delaying the onset and progression of sarcopenia in older adults, improving their health status, and alleviating the burden on healthcare systems.

In recent years, the association between lipid metabolism disorders and muscle dysfunction has garnered increasing attention from the scientific community. Substantial research has confirmed that abnormal lipid metabolism constitutes a significant risk factor for the onset and progression of sarcopenia. Conventionally, the following lipid markers have been utilized: total cholesterol (TC), triglycerides (TG), high-density lipoprotein cholesterol (HDL-C), and low-density lipoprotein cholesterol (LDL-C). In the extant literature, there is evidence to suggest a correlation between reduced HDL-C levels and increased sarcopenia risk. However, the association between atherosclerotic lipid markers, such as LDL-C and TG, and sarcopenia remains controversial (4–6). It is important to note that individual lipid markers reflect only specific aspects of lipid metabolism. As a result, they cannot comprehensively or accurately represent the overall metabolic balance, thus limiting their predictive value for sarcopenia risk. The non-high-density lipoprotein cholesterol to high-density lipoprotein cholesterol ratio (NHHR) has been identified as a novel comprehensive lipid indicator. It has been demonstrated that this approach more accurately reflects the equilibrium between atherogenic and protective lipids, thereby enabling a more precise assessment of the degree of lipid metabolism disorder (7).

The extant research suggests that NHHR exhibits superior predictive value in comparison to individual lipid markers in the forecasting of the risk of multiple chronic conditions, including cardiovascular disease, metabolic syndrome, and diabetes. Elevated levels of NHHR have been demonstrated to be closely associated with increased cardiovascular disease incidence and poor prognosis (7–9). However, to date, studies investigating the association between NHHR and sarcopenia in the elderly remain scarce, leaving the relationship between the two unclear. It is evident that HDL-C provides protection to skeletal muscle cells via multiple pathways, including reverse cholesterol transport, anti-inflammatory and antioxidant effects, regulation of endothelial function, and inhibition of muscle cell apoptosis (10). In contrast, excessive non-HDL-C has been shown to impair mitochondrial function in skeletal muscle cells through lipotoxicity, induce muscle cell apoptosis, and inhibit muscle protein synthesis, consequently resulting in reduced muscle mass and diminished muscle function (11). Consequently, this study employs a combined cross-sectional and prospective cohort approach to systematically investigate the association between NHHR and sarcopenia in the elderly adults, along with its dose-response relationship. Subgroup analyses will validate the robustness of these associations, with the aim of addressing current research gaps and providing novel insights and theoretical foundations for risk assessment and clinical intervention in sarcopenia among the elderly adults.

## Materials and methods

### Data source and study population

**FIGURE 1:**
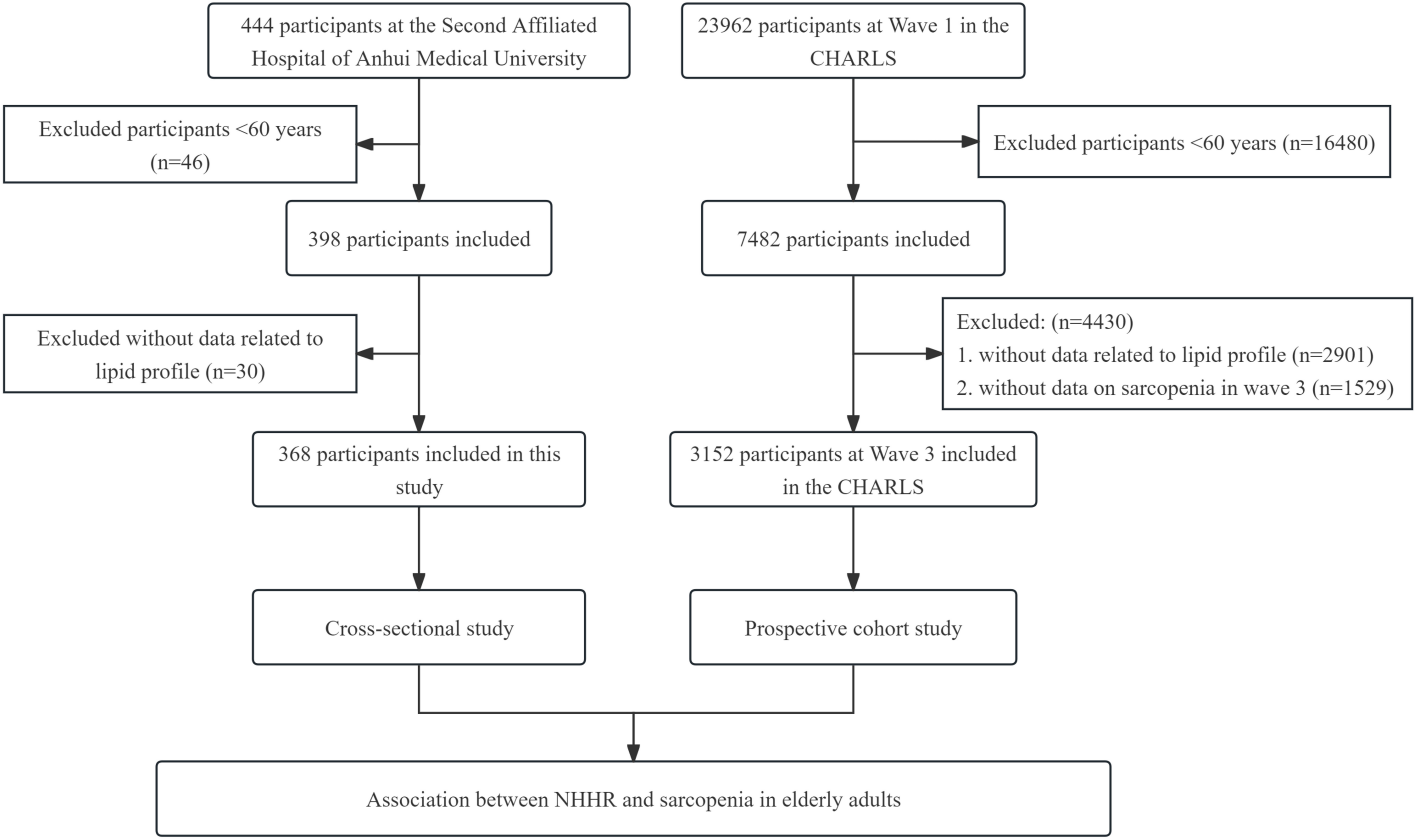
Flowchart of the population included in our study.

The study subjects for this cross-sectional study were elderly adults admitted to the Second Affiliated Hospital of Anhui Medical University between April 2020 and June 2025. All participants signed written informed consent forms. This study adheres to the ethical principles of the Declaration of Helsinki and has been approved by the Ethics Committee of the Second Affiliated Hospital of Anhui Medical University (Approval No.: 2020-ky-012). Exclusion criteria: (1) Age < 60 years; (2) Lack of lipid profile data precluding NHHR calculation; (3) Presence of severe hepatic or renal impairment, malignancy, acute infection, autoimmune disease, thyroid dysfunction, or other conditions potentially affecting muscle or lipid metabolism; (4) Severe neurological disorders precluding completion of muscle function assessment; (5) Hormone therapy, nutritional supplement interventions, or regular resistance exercise training within the preceding 3 months, potentially affecting muscle mass and function; (6) Severe cognitive impairment, psychiatric disorders, or refusal to cooperate with study procedures. Following rigorous screening, 368 participants were ultimately enrolled: 187 males and 181 females, with a mean age of 67.52 ± 5.83 years (Figure 1).

The prospective cohort study data were derived from the China Health and Retirement Longitudinal Study (CHARLS). This study selected data from the first wave (2011–2012) of CHARLS as baseline information and data from the third wave (2015–2016) as follow-up endpoint information, screening participants who met the inclusion criteria. Exclusion criteria: (1) age < 60 years; (2) absence of baseline lipid profile data precluding NHHR calculation; (3) pre-existing diagnosis of sarcopenia at baseline; (4) failure to complete Wave 3 follow-up or unavailability of sarcopenia assessment data at follow-up endpoint. Following screening, 3,152 participants were ultimately included: 1,471 males (46.67%) and 1,681 females (53.33%). The mean baseline age was 66.88 ± 5.75 years, with a median follow-up duration of 4.5 years (Figure 1).

### Sarcopenia

The diagnosis of sarcopenia employs the 2019 Asian Working Group for Sarcopenia (AWGS) criteria: appendicular skeletal muscle mass (ASM) measured by dual-energy X-ray absorptiometry (DXA) must be less than 7.0 kg/m^2^ for males or less than 5.4 kg/m^2^ for females (12).

### Potential covariates

Covariates included gender, age, marital status, educational attainment, smoking status, and alcohol consumption status. The clinical indicators encompassed waist circumference (WC), height, body mass index (BMI), hypertension, diabetes mellitus, heart disease, and stroke. The biochemical indicators encompassed fasting blood glucose (FBG), glycated hemoglobin (HbA1c), HDL-C, LDL-C, TG, TC, creatinine, blood urea nitrogen (BUN) and uric acid (UA), all measured via fully automated biochemical analyzers.

### Calculation of indicators

NHHR is calculated using the formula: NHHR = (TC − HDL-c)/HDL-c.

Triglyceride glucose (TyG) index = ln (TG (mg/dL)) × FBG (mg/dL)/2.

TyG-BMI index = TyG × BMI (kg/m^2^).

TyG-WC index = TyG × WC (cm).

TyG-WHtR index = TyG × (WC (cm) /height (cm)).

TyG-ABSI index = TyG × WC (m)/(BMI^∧^(2/3) × height^∧^(1/2)).

### Statistical analysis

All data were subjected to statistical analysis using R 4.2. The normality of all data was tested. Continuous variables that followed a normal distribution were expressed as mean ± standard deviation ($\bar{x}$ ± s) and then subjected to comparison using an independent samples *t*-test. Variables that did not adhere to a normal distribution are expressed as the median (interquartile range) and were subjected to comparison using non-parametric tests. Categorical variables were expressed as numbers and percentages, and comparisons between groups were made using the chi-squared test or Fisher’s exact test. Several multiple logistic regression and Cox proportional hazards regression models were developed to investigate the relationship between NHHR and the prevalence of sarcopenia. Restricted cubic spline (RCS) plots were used to further investigate the nonlinear relationship between NHHR and sarcopenia prevalence. All continuous variables were first cleaned of extreme outliers using the interquartile range (IQR) method, and the source of any samples whose values on the x-axis of the RCS plot fell outside the normal physiological range was specified. In addition, subgroup analyses were also performed in this study. Mediation analysis was employed to examine the mediating effects of TyG index, TyG-BMI, TyG-WC, TyG-WHtR and TyG-ABSI between NHHR and sarcopenia. For the mediation analysis, the Bootstrap method was employed, with 5,000 repeated random samples taken to calculate the adjusted 95% confidence intervals for the direct effect, indirect effect and total effect; all analyses were adjusted for several confounding factors to verify the stability of the mediation effect. If the 95% confidence interval for the indirect effect did not include 0, the mediation effect was deemed statistically significant. All statistical tests were two-tailed, with *P* < 0.05 indicating statistically significant differences.

## Results

### Baseline characteristics of cross-sectional study participants

The cross-sectional component of the study involved 368 elderly adults, of whom 147 cases of sarcopenia were identified, yielding a prevalence rate of 40.0%. The results demonstrated that, in comparison with the non-sarcopenic group, the sarcopenic group exhibited significantly lower BMI levels, markedly reduced TG levels, significantly elevated HDL-c levels, and markedly increased NHHR levels. Concurrently, the proportion of males in the sarcopenic group was significantly higher than in the non-sarcopenic group (*P* < 0.05). No statistically significant differences were observed between the two groups regarding age, FBG, TC, LDL-c, creatinine, UA, HbA1c, or the prevalence of hypertension, heart disease, and stroke (*P* > 0.05). The application of quartile analysis to the NHHR revealed a progressive decrease in the prevalence of sarcopenia among elderly adults, with increasing NHHR quartile levels (*P* = 0.033) (Table 1).

**TABLE 1:** Participants demographics and baseline characteristics.

Variables	Total (*n* = 368)	Without sarcopenia (*n* = 221)	With sarcopenia (*n* = 147)	*P*
Age (y)	67.83 ± 5.74	67.66 ± 5.87	68.10 ± 5.55	0.478
BMI (Kg/m^2^)	24.41 ± 3.15	25.20 ± 3.15	23.21 ± 2.75	< 0.001
FBG (mmol/L)	7.97 ± 3.07	7.76 ± 2.93	8.28 ± 3.24	0.114
TC (mmol/L)	4.54 ± 1.20	4.62 ± 1.26	4.43 ± 1.09	0.149
HDL-c (mmol/L)	1.12 ± 0.29	1.09 ± 0.27	1.17 ± 0.30	0.007
LDL-c (mmol/L)	2.76 ± 0.89	2.79 ± 0.91	2.71 ± 0.85	0.398
TG (mmol/L)	1.84 ± 1.59	2.03 ± 1.82	1.56 ± 1.11	0.005
Creatine (mmol/L)	70.74 ± 29.73	69.29 ± 30.13	72.93 ± 29.07	0.253
UA (mmol/L)	302.08 ± 93.28	301.19 ± 95.12	303.42 ± 90.73	0.825
HbA1c (%)	8.70 ± 2.06	8.69 ± 2.03	8.73 ± 2.12	0.868
NHHR	3.21 ± 1.23	3.40 ± 1.31	2.93 ± 1.04	< 0.001
Gender, n(%)				< 0.001
Male	181 (49.18)	90 (40.72)	91 (61.90)	
Female	187 (50.82)	131 (59.28)	56 (38.10)	
Hypertension, n(%)	229 (62.23)	140 (63.35)	89 (60.54)	0.587
Heart disease, n(%)	60 (16.30)	32 (14.48)	28 (19.05)	0.245
Stroke, n(%)	74 (20.11)	42 (19.00)	32 (21.77)	0.517
NHHR quartile, n(%)				0.033
Q1	92 (25.00)	45 (20.36)	47 (31.97)	
Q2	92 (25.00)	53 (23.98)	39 (26.53)	
Q3	92 (25.00)	60 (27.15)	32 (21.77)	
Q4	92 (25.00)	63 (28.51)	29 (19.73)	

BMI, body mass index; FBG, fasting blood glucose; HbA1c, glycated hemoglobin; TC, total cholesterol; TG, triglyceride; HDL-c, high-density lipoprotein cholesterol; LDL-c, low-density lipoprotein cholesterol; UA, uric acid; NHHR, non-high-density lipoprotein cholesterol to high-density lipoprotein cholesterol ratio.

### Logistic regression analysis of the association between NHHR and sarcopenia in elderly adults

The application of a logistic regression analysis yielded a significant negative correlation between NHHR and sarcopenia risk (Table 2). In comparison with the lowest quartile (Q1) of NHHR, Q3 (OR = 0.51–0.52, 95% CI: 0.27–0.99, *P* = 0.026–0.045) and Q4 (OR = 0.44–0.45, 95% CI: 0.23–0.86, *P* = 0.007–0.016) were found to be significantly associated with reduced sarcopenia risk in both unadjusted (Model 1) and stepwise adjusted models (Models 2–4). The Q2 showed no significant association in comparison with Q1 (OR = 0.70–0.84, 95% CI: 0.39–1.56, *P* = 0.238–0.580). Following the adjustment of confounding factors, a unit increase in NHHR was observed to be associated with a 31% reduction in sarcopenia risk (OR = 0.69, 95% CI: 0.56–0.86, *P* < 0.001).

**TABLE 2:** Cross-sectional association between NHHR and sarcopenia.

Variables	Model 1		Model 2		Model 3		Model 4	
	OR (95%CI)	*P*	OR (95%CI)	*P*	OR (95%CI)	*P*	OR (95%CI)	*P*
NHHR								
Q1	1.00 (Reference)		1.00 (Reference)		1.00 (Reference)		1.00 (Reference)	
Q2	0.70 (0.39–1.26)	0.238	0.73 (0.40–1.33)	0.308	0.74 (0.41–1.35)	0.329	0.84 (0.45–1.56)	0.580
Q3	0.51 (0.28–0.92)	0.026	0.51 (0.27–0.93)	0.029	0.52 (0.28–0.95)	0.035	0.52 (0.28–0.99)	0.045
Q4	0.44 (0.24–0.80)	0.007	0.44 (0.24–0.81)	0.009	0.45 (0.24–0.84)	0.012	0.45 (0.23–0.86)	0.016
NHHR	0.70 (0.58–0.86)	< 0.001	0.70 (0.57–0.86)	< 0.001	0.71 (0.58–0.87)	< 0.001	0.69 (0.56–0.86)	< 0.001

Model1, Crude; Model 2, Adjust: gender, age; Model 3, Adjust: gender, age, hypertension, heart diaease, stroke. Model 4, Adjust: gender, age, hypertension, heart diaease, stroke, FBG, HbA1c, Creatine, UA.

### Subgroup analysis in a cross-sectional study

**FIGURE 2:**
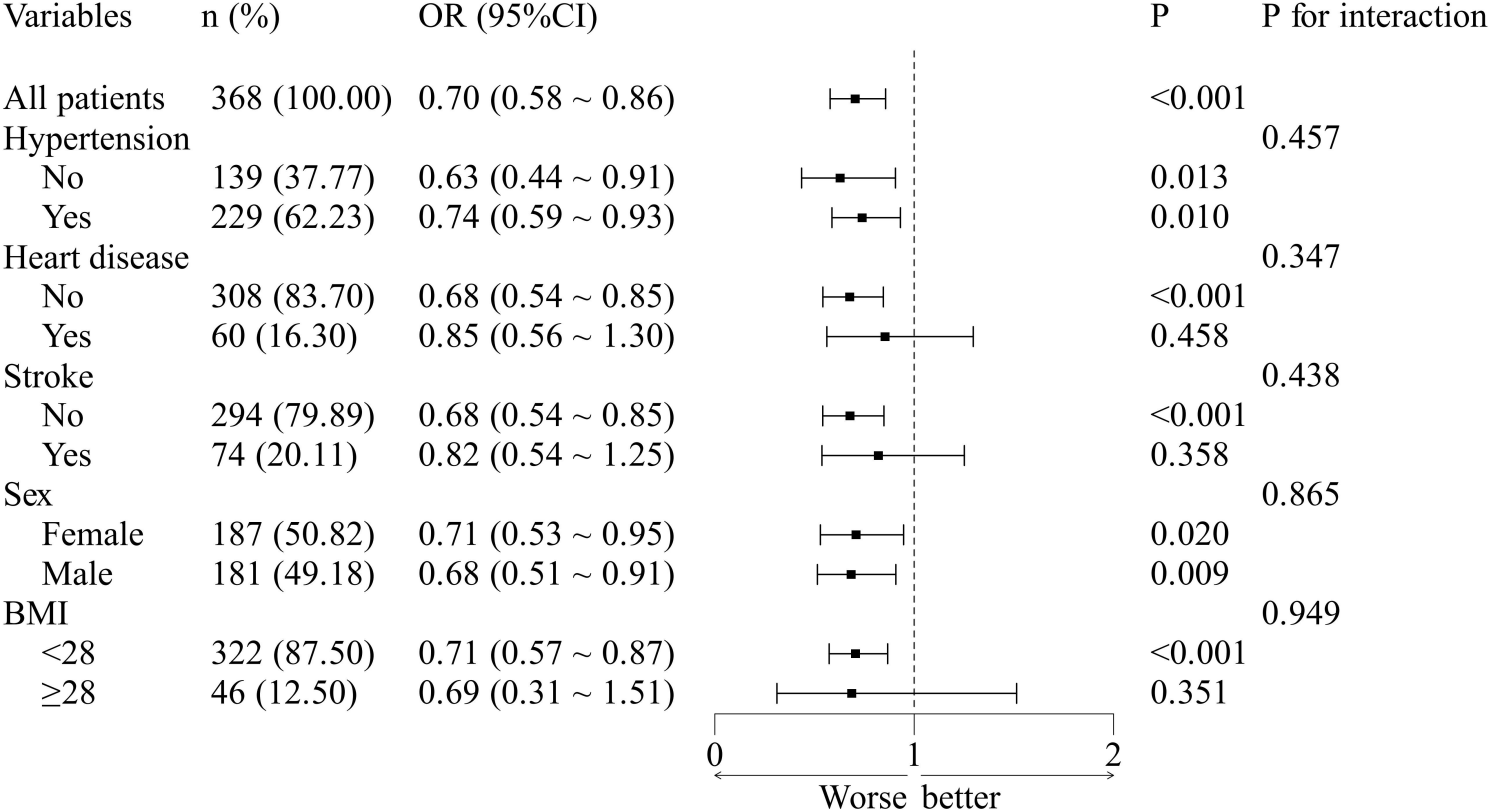
Subgroup analysis of the association between continuous NHHR and sarcopenia risk in cross-sectional study. All P values for interaction were greater than 0.05, indicating no significant effect modification across subgroups.

A subgroup analysis was conducted, with gender, BMI, hypertension, heart disease and stroke designated as the subgroups for the purpose of examining potential influencing factors. Overall, each one-unit increment in NHHR was associated with a 30% decreased odds of sarcopenia (OR = 0.70, 95%CI 0.58–0.86, *P* < 0.001). This inverse relationship was statistically significant in participants with or without hypertension, those free of heart disease or stroke, both sexes, and subjects with BMI < 28 kg/m^2^. No significant correlation was detected among patients complicated with heart disease, stroke or BMI ≥ 28 kg/m^2^, largely owing to small sample sizes in these subsets. Interaction tests yielded all *P* > 0.05, confirming no remarkable subgroup heterogeneity (Figure 2).

### Dose-response analysis in a cross-sectional study

**FIGURE 3:**
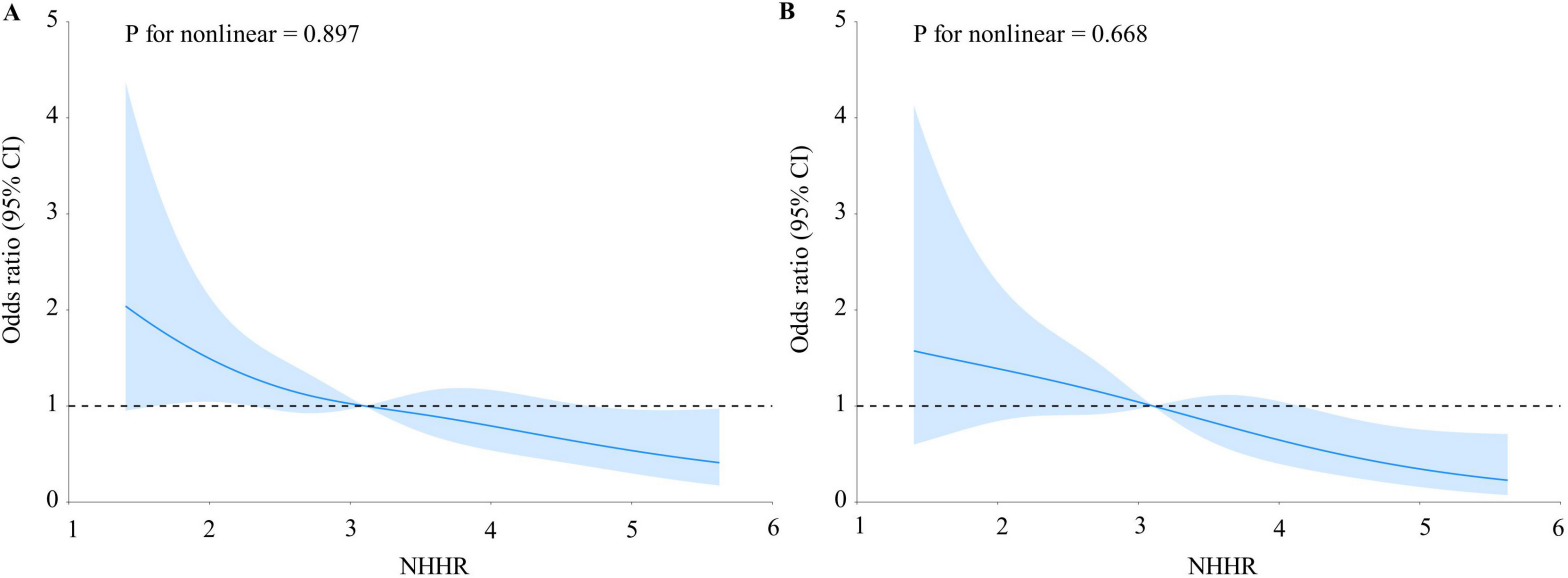
Dose-response analysis in a cross-sectional study. **(A)** Nonlinear correlation analysis between NHHR and sarcopenia, unadjusted for confounding factors. **(B)** Nonlinear correlation analysis between NHHR and sarcopenia, adjusting for confounding factors. The adjusted confounding factors include: gender, age, hypertension, heart disease, stroke, FBG, HbA1c, creatinine, UA.

Cross-sectional RCS analysis revealed a clear linear association between NHHR and sarcopenia in elderly adults (P for nonlinear = 0.897), indicating a sustained linear decline in sarcopenia risk with increasing NHHR levels (Figure 3A). This linear relationship was found to persist even after adjustments were made for multiple confounding factors (P for nonlinear = 0.668) (Figure 3B).

### Baseline characteristics of prospective study participants

A prospective cohort study was conducted, enrolling 3,152 elderly individuals without baseline sarcopenia, with a mean age of 66.88 ± 5.75 years. The cohort comprised 1,471 males (46.67%) and 1,681 females (53.33%). A comparative analysis of baseline characteristics across NHHR quartile groups revealed that there was a progressive increase in FBG, TC, LDL-c, TG, and HbA1c with ascending NHHR quartiles, whilst HDL-c levels exhibited a progressive decrease (*P* < 0.001). Concurrently, there was a progressive increase in the prevalence of diabetes, hypertension, cardiovascular disease and stroke with rising NHHR quartiles (*P* < 0.05), whilst smoking and alcohol consumption rates showed a progressive decline (*P* < 0.001). No statistically significant differences were observed across NHHR quartiles regarding gender, age, BMI, marital status, or educational attainment (P > 0.05). During the subsequent follow-up period, 107 cases of sarcopenia were documented among 3,152 subjects, resulting in a cumulative incidence rate of 3.39%. The incidence of sarcopenia exhibited a significant downward trend with increasing NHHR quartile levels (P for trend < 0.001) (Table 3).

**TABLE 3:** The baseline characteristics are based on quartiles of the NHHR in CHARLS.

Variables	Total (*n* = 3152)	Q1 (*n* = 788)	Q2 (*n* = 788)	Q3 (*n* = 788)	Q4 (*n* = 788)	*P* for trend
Age (y)	66.88 ± 5.75	66.94 ± 5.62	67.02 ± 6.12	66.60 ± 5.60	66.97 ± 5.65	0.468
BMI (Kg/m^2^)	23.28 ± 3.74	21.55 ± 3.32	22.68 ± 3.62	23.88 ± 3.75	24.85 ± 3.42	< 0.001
FBG (mg/dL)	111.89 ± 35.76	104.82 ± 25.38	107.33 ± 29.43	111.50 ± 32.22	123.91 ± 48.52	< 0.001
TC (mmol/L)	5.06 ± 1.01	4.55 ± 0.87	4.91 ± 0.86	5.16 ± 0.87	5.63 ± 1.11	< 0.001
HDL-c (mmol/L)	1.33 ± 0.40	1.74 ± 0.39	1.43 ± 0.26	1.21 ± 0.21	0.95 ± 0.21	< 0.001
LDL-c (mmol/L)	3.10 ± 0.92	2.51 ± 0.63	3.03 ± 0.68	3.34 ± 0.76	3.51 ± 1.19	< 0.001
TG (mmol/L)	1.47 ± 1.14	0.88 ± 0.37	1.14 ± 0.45	1.43 ± 0.60	2.42 ± 1.77	< 0.001
BUN (mmol/L)	16.26 ± 4.51	16.56 ± 4.71	16.64 ± 4.76	15.85 ± 4.21	16.00 ± 4.27	< 0.001
Creatine (mmol/L)	0.80 ± 0.20	0.78 ± 0.19	0.81 ± 0.22	0.79 ± 0.18	0.83 ± 0.22	< 0.001
UA (mmol/L)	4.55 ± 1.27	4.31 ± 1.18	4.50 ± 1.30	4.56 ± 1.22	4.83 ± 1.32	< 0.001
HbA1c (%)	5.31 ± 0.80	5.17 ± 0.58	5.24 ± 0.61	5.30 ± 0.70	5.54 ± 1.15	< 0.001
Diabetes, n(%)	528 (16.75)	83 (10.53)	102 (12.94)	132 (16.75)	211 (26.78)	< 0.001
Gender, n(%)						0.046
Male	1471 (46.67)	400 (50.76)	367 (46.57)	356 (45.18)	348 (44.16)	
Female	1681 (53.33)	388 (49.24)	421 (53.43)	432 (54.82)	440 (55.84)	
Drinking, n(%)	949 (30.11)	297 (37.69)	246 (31.22)	200 (25.38)	206 (26.14)	< 0.001
Smoking, n(%)	919 (29.16)	263 (33.38)	239 (30.33)	226 (28.68)	191 (24.24)	< 0.001
Hypertension, n(%)	1586 (50.32)	314 (39.85)	400 (50.76)	409 (51.90)	463 (58.76)	< 0.001
Marry, n(%)						0.749
Married or partnered	2493 (79.09)	616 (78.17)	618 (78.43)	629 (79.82)	630 (79.95)	
Other status	659 (20.91)	172 (21.83)	170 (21.57)	159 (20.18)	158 (20.05)	
Education, n(%)						0.282
Non-former education	1171 (37.15)	296 (37.56)	317 (40.23)	291 (36.93)	267 (33.88)	
Primary school	1461 (46.35)	368 (46.70)	347 (44.04)	367 (46.57)	379 (48.10)	
Secondary school and above	520 (16.50)	124 (15.74)	124 (15.74)	130 (16.50)	142 (18.02)	
Heart disease, n(%)	482 (15.29)	114 (14.47)	107 (13.58)	113 (14.34)	148 (18.78)	0.017
Stroke, n(%)	90 (2.86)	13 (1.65)	22 (2.79)	21 (2.66)	34 (4.31)	0.016
Sarcopenia, n(%)	107 (3.39)	50 (6.35)	29 (3.68)	19 (2.41)	9 (1.14)	< 0.001

BMI, body mass index; FBG, fasting blood glucose; HbA1c, glycated hemoglobin; TC, total cholesterol; TG, triglyceride; HDL-c, high-density lipoprotein cholesterol; LDL-c, low-density lipoprotein cholesterol; BUN, blood urea nitrogen; UA, uric acid; NHHR, non-high-density lipoprotein cholesterol to high-density lipoprotein cholesterol ratio.

### Cox regression analysis of the association between NHHR and sarcopenia in elderly adults

The relationship between NHHR and the risk of sarcopenia was investigated through the utilization of Cox proportional hazards regression models. The robustness of the findings was validated through the implementation of four progressively adjusted models. The findings of the study demonstrated that, in comparison with the lowest quartile (Q1) of NHHR, the risk of sarcopenia exhibited a significant decreasing trend with increasing NHHR levels (P < 0.05 in all models). In the final adjusted Model 4, the HR and 95% CI for sarcopenia occurrence in the Q2, Q3, and Q4 groups were 0.58 (0.36–0.94, P = 0.027), 0.43 (0.25–0.73, *P* = 0.002), and 0.21 (0.10–0.43, *P* < 0.001), respectively. This inverse association remained consistent across all adjusted models (Table 4). Following the adjustment of confounding factors, a unit increase in NHHR was observed to be associated with a 37% reduction in sarcopenia risk (HR = 0.63, 95% CI: 0.51–0.77, *P* < 0.001) (Table 4).

**TABLE 4:** Results of cox regression analysis between NHHR and sarcopenia.

Variables	Model 1		Model 2		Model 3		Model 4	
	HR (95%CI)	*P*	HR (95%CI)	*P*	HR (95%CI)	*P*	HR (95%CI)	*P*
NHHR								
Q1	1.00 (Reference)		1.00 (Reference)		1.00 (Reference)		1.00 (Reference)	
Q2	0.57 (0.36–0.91)	0.018	0.57 (0.36–0.90)	0.016	0.60 (0.38–0.96)	0.033	0.58 (0.36–0.94)	0.027
Q3	0.37 (0.22–0.63)	< 0.001	0.41 (0.24–0.70)	< 0.001	0.43 (0.25–0.74)	0.002	0.43 (0.25–0.73)	0.002
Q4	0.18 (0.09–0.36)	< 0.001	0.20 (0.10–0.40)	< 0.001	0.21 (0.10–0.43)	< 0.001	0.21 (0.10–0.43)	< 0.001
NHHR	0.57 (0.46–0.69)	< 0.001	0.60 (0.49–0.73)	< 0.001	0.61 (0.50–0.74)	< 0.001	0.63 (0.51–0.77)	< 0.001

Model 1: Crude; Model 2: Adjust: gender, age; Model 3: Adjust: gender, age, hypertension, heart diaease, stroke. Model 4: Adjust: gender, age, hypertension, heart diaease, stroke, FBG, HbA1c, Creatine, UA.

### Subgroup analysis in a prospective study

**FIGURE 4:**
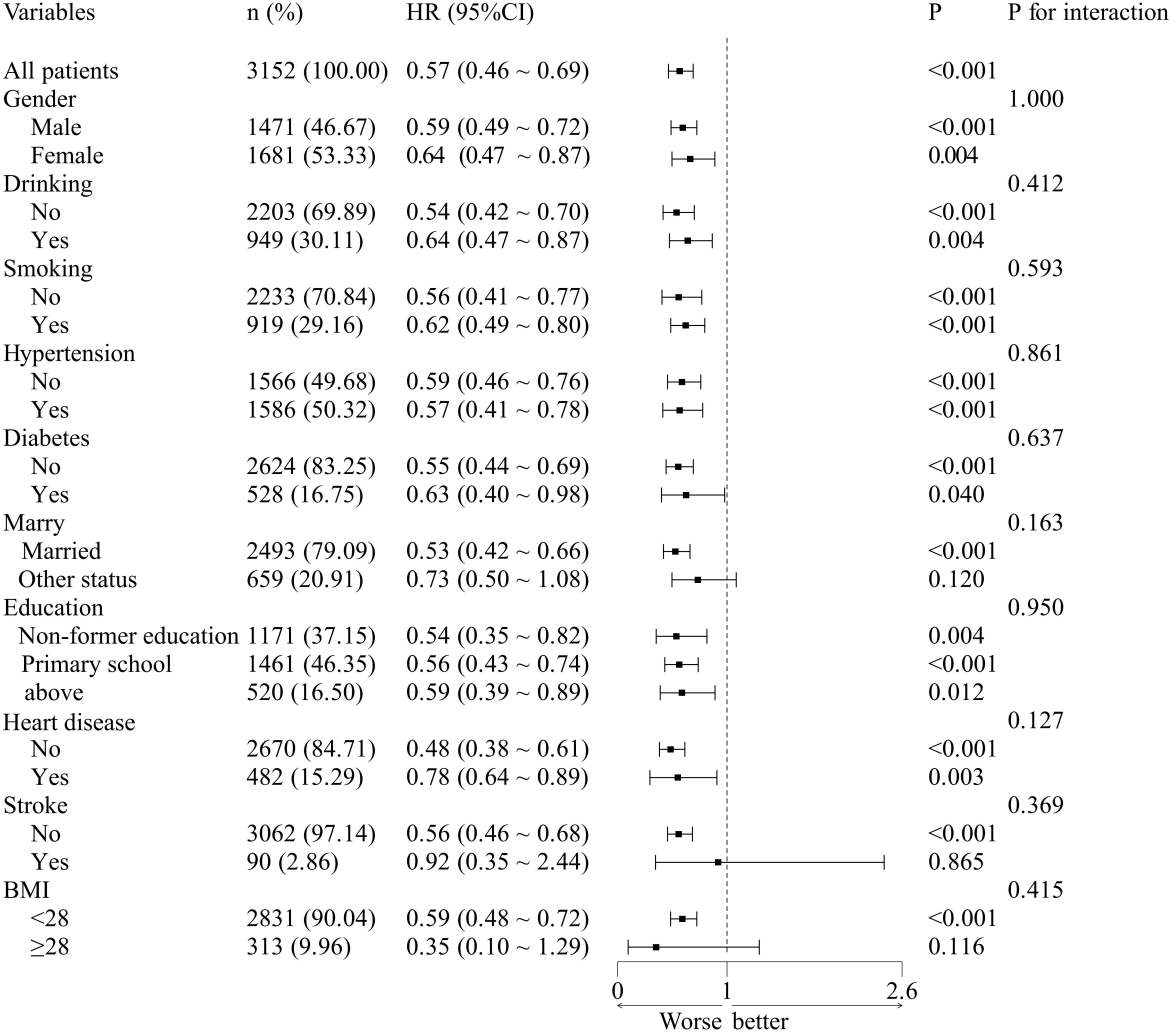
Stratified Cox regression subgroup analysis of continuous NHHR and incident sarcopenia in prospective cohort study. No statistically significant interaction was detected in all stratification variables.

Stratified Cox models revealed that per unit increase of NHHR independently lowered the hazard of new-onset sarcopenia in the whole cohort (HR = 0.57, 95%CI 0.46–0.69, *P* < 0.001). The protective effect persisted across most subgroups classified by demographic characteristics and chronic conditions. Comparatively, subgroups with diabetes, unmarried status, heart disease, stroke and BMI ≥ 28 kg/m^2^ failed to reach statistical significance. No significant interaction existed for all stratification factors (all P for interaction > 0.05), supporting the consistency of this predictive association across diverse populations (Figure 4).

### Dose-response analysis in a prospective study

**FIGURE 5:**
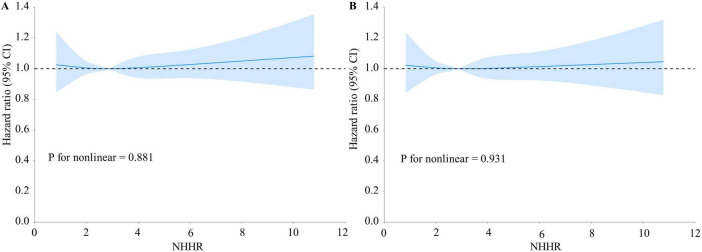
Dose-response analysis in a prospective study. **(A)** Nonlinear correlation analysis between NHHR and sarcopenia, unadjusted for confounding factors. **(B)** Nonlinear correlation analysis between NHHR and sarcopenia, adjusting for confounding factors. The adjusted confounding factors include: gender, age, hypertension, heart disease, stroke, FBG, HbA1c, creatinine, UA. Extreme outliers have been removed using the interquartile range method; the range on the horizontal axis covers all valid NHHR measurements from the study participants. Although some values fall outside the standard reference range for the general population, they are all genuine and valid data obtained after outliers have been removed.

Prospective RCS analysis revealed a clear linear association between NHHR and sarcopenia in elderly adults (P for nonlinear = 0.881), indicating a sustained linear decline in sarcopenia risk with increasing NHHR levels (Figure 5A). This linear relationship was found to persist even after adjustments were made for multiple confounding factors (P for nonlinear = 0.931) (Figure 5B). A small number of elderly study participants exhibited elevated NHHR levels due to severe dyslipidemia; these were included in the analysis after outlier handling, and the linear association trend was not affected by these extreme values.

### Mediation analysis

**FIGURE 6:**
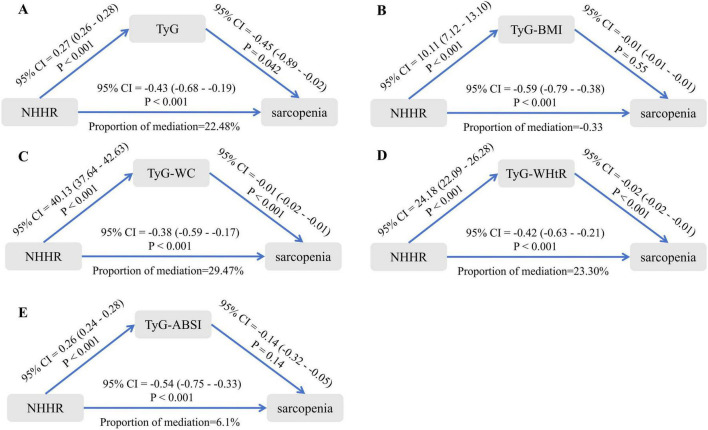
Mediation analysis results of TyG-related indices on the association between NHHR and sarcopenia among the Chinese elderly adults. **(A)** TyG. **(B)** TyG-BMI. **(C)** TyG-WC. **(D)** TyG-WHtR. **(E)** TyG-ABSI. The adjusted confounding factors include: gender, age, hypertension, heart disease, stroke, FBG, HbA1c, creatinine, UA.

After adjusting for several confounding factors, mediation analysis revealed significant negative total effects between TyG, TyG-BMI, TyG-WC, TyG-WHtR, TyG-ABSI, and sarcopenia (all *P* < 0.001). The mediation proportions for TyG, TyG-WC, and TyG-WHtR were 22.48, 29.47, and 23.30% respectively (all *P* < 0.001). No significant mediating effects were observed for TyG-BMI and TyG-ABSI (*P* > 0.05) (Figure 6).

## Discussion

The present study adopted a dual-study design combining cross-sectional and prospective cohort analyses to systematically investigate the association and dose-response relationship between NHHR and sarcopenia in the Chinese elderly population, providing supplementary prospective evidence in this field. The findings suggest that NHHR in the elderly population displays a stable, linear, negative dose-response relationship with the risk of sarcopenia. This association remains consistent across different subgroups, and TyG-related indices play a partial mediating role in this relationship. These findings suggest that NHHR, as a convenient and accessible composite indicator of lipid metabolism, may serve as a potential biomarker for the early risk stratification of sarcopenia in elderly adults. This inverse association should be interpreted with caution, as it may also reflect differences in nutritional status, body composition, lipid reserve, and the lipid paradox commonly observed in older and frail populations, rather than a direct protective effect of NHHR against sarcopenia.

It is hypothesized that the negative correlation between NHHR and sarcopenia in the elderly may be mediated through several interconnected mechanisms, based on existing research reports and the findings of this study. These mechanisms interact synergistically to regulate the balance between skeletal muscle synthesis and degradation, thereby influencing the onset and progression of sarcopenia. Firstly, the protective role of HDL-C constitutes a key mechanism underpinning this association. As the sole protective lipid component in the body, HDL-c possesses multiple physiological functions, with its protective role for skeletal muscle cells gaining increasing attention (10). The function of HDL-C is to facilitate the reverse transport of cholesterol, thereby transferring excess cholesterol from skeletal muscle cells to the liver for subsequent metabolism and excretion. This process prevents the accumulation of abnormal cholesterol within muscle cells, thereby reducing the risk of cellular damage. Concurrently, HDL-c has been shown to possess potent anti-inflammatory and antioxidant properties, including the capacity to scavenge reactive oxygen species within skeletal muscle cells. In addition, it has been observed to suppress inflammatory cytokine expression and to alleviate chronic inflammatory responses in muscle tissue. Chronic inflammation has been identified as a key driver in the progression of sarcopenia, contributing to reduced muscle mass and diminished function through inducing muscle cell apoptosis and inhibiting muscle protein synthesis (5). Furthermore, HDL-C has been demonstrated to enhance skeletal muscle blood supply by regulating endothelial function, thereby ensuring the delivery of sufficient oxygen and nutrients to muscle cells, thus promoting muscle protein synthesis and maintaining stable muscle function. However, a number of studies have indicated a correlation between extremely high HDL-C levels and an increased risk of sarcopenia (11, 13). The present study demonstrated that HDL-c levels were significantly higher in the sarcopenia group than in the non-sarcopenia group, with NHHR levels also markedly elevated. This finding suggests that increased HDL-C levels may be a significant factor contributing to the negative correlation between NHHR and sarcopenia.

Secondly, the dual role of non-HDL-C may also modulate the association between the two. It has been established that elevated levels of non-HDL-C have the potential to induce cellular damage to skeletal muscle cells, a process that is attributed to the toxicity of lipids (14). In cases where non-HDL-C levels are elevated to a significant degree, an accumulation of lipids occurs within the skeletal muscle cells in an atypical manner. This phenomenon results in a state of mitochondrial dysfunction. This has been shown to impair energy metabolism within muscle cells while simultaneously inducing apoptosis and inhibiting muscle protein synthesis, thereby increasing the risk of sarcopenia. However, moderate non-HDL-C is essential for normal skeletal muscle cell physiology, providing energy substrates for muscle contraction and anabolic processes. The NHHR is predicated on the integration of the equilibrium between non-HDL-C and HDL-C. Elevated NHHR levels may indicate a predominance of HDL-c’s protective effects or an optimal non-HDL-c concentration. At this stage, the metabolic processes associated with lipids are in a state of equilibrium, thus enabling the sustenance of optimal skeletal muscle structure and function. This, in turn, serves to mitigate the likelihood of sarcopenia.

Furthermore, the interaction between lipid metabolism and insulin resistance may represent an additional potential mechanism underpinning their association. Research indicates that elevated NHHR levels correlate closely with improved insulin sensitivity, whilst insulin resistance constitutes a significant risk factor for the development of sarcopenia (15, 16). Insulin performs a dual function in the human body, regulating glucose metabolism while also promoting muscle protein synthesis and inhibiting muscle protein breakdown. This vital role of insulin in maintaining skeletal muscle mass and function is well-documented. The development of insulin resistance results in a diminution of the actions of insulin, consequently leading to a reduction in muscle protein synthesis and an increase in muscle protein breakdown. This, in turn, has been shown to result in a decrease in muscle mass and a decline in muscle function, ultimately leading to the development of sarcopenia. In this study, as NHHR levels increased, both FBG and HbA1c levels rose progressively, yet the risk of sarcopenia gradually decreased. This finding indicates that the inverse association between NHHR and sarcopenia may be partially explained by improved insulin sensitivity correlated with NHHR, which may counteract the adverse impact of elevated blood glucose on muscle function. This observational result does not confirm a direct protective effect of NHHR on skeletal muscle. Mediation analysis results indicate that TyG, TyG-WC, and TyG-WHtR exert significant mediating effects between NHHR and sarcopenia. This suggests that insulin resistance may act as a key pathway underlying the inverse association between NHHR and sarcopenia risk.

From a broader perspective, NHHR reflects an overall imbalance between atherogenic apoB-containing lipoprotein particles and HDL-mediated protective pathways, rather than merely a numerical ratio of cholesterol components. Elevated triglyceride levels, which drive the increase of non-HDL components, are core markers of global lipoprotein derangement, including the accumulation of triglyceride-rich lipoproteins, remnant cholesterol, small dense low-density lipoprotein particles, and functional remodeling of HDL particles (17). This integrated lipoprotein phenotype is closely linked to systemic metabolic dysregulation, which may jointly influence skeletal muscle homeostasis through multiple pathways beyond insulin resistance alone. This also explains why TyG-related indices, which integrate triglyceride and glucose levels, can partially mediate the association between NHHR and sarcopenia, as they capture a wider cardiometabolic risk profile rather than insulin resistance exclusively.

Furthermore, chronic low-grade inflammation is a core driver of sarcopenia progression, and the association between lipid metabolism and muscle health cannot be fully interpreted without considering the lipoinflammatory background. Atherogenic lipid imbalance can trigger and amplify chronic inflammatory responses in muscle tissue, which in turn exacerbate muscle protein breakdown and impair muscle regeneration. Inflammatory biomarkers such as the neutrophil-to-lymphocyte ratio have been shown to correlate with subclinical atherosclerosis and provide incremental prognostic value beyond traditional lipid parameters (18), supporting that NHHR acts within a broader cardiometabolic and inflammatory risk framework to modulate sarcopenia risk.

At present, there is no international consensus on the association between NHHR and sarcopenia; the findings of this study differ from those of some studies conducted on European and American populations, and this heterogeneity can be explained by four factors. Firstly, there are differences in the ethnicity and baseline characteristics of the study populations. The majority of extant studies reporting a positive association have been conducted among adult populations in the United States (19–21). In these populations, the prevalence of obesity, baseline lipid levels and baseline muscle mass were all significantly higher than in the Chinese elderly population. Furthermore, some studies included middle-aged individuals, whereas sarcopenia is a condition prevalent among the elderly, and differences in age composition can directly influence the direction of the association. Studies focusing on the elderly population in the United States have similarly concluded that there is a negative association (22), consistent with the findings of this study, suggesting that age stratification is a key factor influencing the association between the two. Secondly, there is a lack of uniformity in the diagnostic criteria for sarcopenia. Research conducted in Europe and the United States primarily utilizes the European Working Group on Sarcopenia (EWGSOP) criteria, which employ higher cut-off values for muscle mass. The present study scrupulously followed the AWGS diagnostic criteria, which are adapted to the physical characteristics of Asian populations; discrepancies in diagnostic cut-off values directly impact estimates of prevalence and the strength of the association. Thirdly, there are discrepancies in study design and exposure measurement. The majority of international studies utilize a cross-sectional design, which is only capable of reflecting associations at a single point in time. The present study incorporated prospective follow-up data spanning 4.5 years, enabling verification of the predictive value of baseline NHHR for the onset of sarcopenia and providing stronger evidence of causal temporal relationships. Fourthly, there are discrepancies in the methodologies employed for adjusting for confounding factors. A number of studies have been conducted that have made adjustments to the data solely for basic demographic indicators. However, these studies have not adequately accounted for confounding factors such as blood glucose levels, renal function and the presence of chronic diseases. The present study implemented a stepwise adjustment process for demographic, anthropometric, and biochemical indicators, as well as a history of chronic diseases. This methodological approach ensured more thorough control for confounding. A domestic study of middle-aged individuals based on the CHARLS database also concluded that a negative association exists (23), which is consistent with the findings of this study and further supports the stability of this association in East Asian populations.

From a clinical perspective, the results of this study’s quartile-based stratification analysis suggest that older adults with an NHHR below the first quartile (Q1 < 2.38 in the cross-sectional analysis; Q1 < 2.51 in the cohort analysis) have a significantly increased risk of sarcopenia; this value may therefore serve as a reference cut-off for initial risk stratification. In comparison with conventional sarcopenia-associated biomarkers, such as high-sensitivity C-reactive protein (hs-CRP), albumin and serum creatinine, the NHHR demonstrates a clear advantage in that it can be calculated using only the four routine lipid profile parameters. It is both low-cost and highly accessible in clinical practice, making it suitable for large-scale initial screening of older adults in the community. Nevertheless, when utilized as a solitary indicator, its capacity for prediction is constrained, and it cannot supplant the gold standard evaluation of muscle mass and strength. It is important to acknowledge that the present study is of an observational nature, and thus, it is only able to confirm the association between NHHR and the risk of sarcopenia; it is not able to establish a causal relationship. The findings of the study are intended exclusively to inform the process of risk stratification for screening purposes. They cannot be used to directly infer a clinical conclusion that an elevated NHHR warrants intervention for sarcopenia. Further intervention studies are required to validate the value of lipid regulation in the prevention and treatment of sarcopenia.

The present study also delineates certain limitations that require refinement and improvement in subsequent research. (1) The NHHR was assessed at the baseline only, with a single measurement; there was an absence of repeated lipid profile data at follow-up, resulting in regression dilution bias, which may have led to an underestimation of the strength of the association. Due to the fact that lipid profiles were not measured during the CHARLS follow-up, it was not possible to analyze the relationship between dynamic changes in NHHR and sarcopenia. (2) The database exhibits an absence of standardized information pertaining to diet, physical activity, and the utilization of statins, hormones, or antihypertensive medication. Given the exclusion of these factors from the adjustments, residual confounding bias is present. Despite the robustness of the results being verified through subgroup analyses, it is not possible to completely eliminate confounding factors. (3) It is important to note that, due to the observational nature of this study, it can only demonstrate an association between the variables under investigation. It cannot establish causation; in order to do so, validation via Mendelian randomization or intervention trials is required. (4) The study population consisted exclusively of elderly Chinese individuals; disparities in ethnicity and diagnostic criteria when compared with studies conducted in Europe and the United States limit the generalizability of the conclusions. (5) The predictive performance of NHHR has yet to be compared with that of other markers, such as hs-CRP, albumin and creatinine. Furthermore, the optimal cut-off value for NHHR screening for sarcopenia remains to be calculated. (6) The NHHR levels of some participants exceeded the normal physiological range. Although extreme outliers were excluded, this may still have had a slight influence on the trend of the dose-response curve.

## Conclusion

The present study demonstrates that there is a significant negative linear dose-response relationship between NHHR and the risk of sarcopenia in in Chinese elderly adults; TyG-related indices play a partial mediating role in this association, and this association remains consistent across different subgroups.

## Data Availability

The original contributions presented in the study are included in the article/supplementary material, further inquiries can be directed to the corresponding authors.

## References

[1] Yuan S Larsson SC. Epidemiology of sarcopenia: prevalence, risk factors, and consequences. *Metabolism*. (2023) 144:155533. 10.1016/j.metabol.2023.155533 36907247

[2] Liu D Wang S Liu S Wang Q Che X Wu G. Frontiers in sarcopenia: advancements in diagnostics, molecular mechanisms, and therapeutic strategies. *Mol Aspects Med*. (2024) 97:101270. 10.1016/j.mam.2024.101270 38583268

[3] Coletta G Phillips SM. An elusive consensus definition of sarcopenia impedes research and clinical treatment: a narrative review. *Ageing Res Rev*. (2023) 86:101883. 10.1016/j.arr.2023.101883 36792012

[4] Guo XY Li LW. Hs-CRP/HDL-C ratio as a predictive inflammatory-lipid marker for sarcopenia: evidence from NHANES 2015-2018. *Front Immunol*. (2025) 16:1600421. 10.3389/fimmu.2025.1600421 40672942 PMC12264492

[5] Bi B Dong X Yan M Zhao Z Liu R Li S et al.. Dyslipidemia is associated with sarcopenia of the elderly: a meta-analysis. *BMC Geriatr*. (2024) 24:181. 10.1186/s12877-024-04761-4 38395763 PMC10885450

[6] Yin X Song H Chen H Yang X Zhang T. Association between lipid ratios and sarcopenia and the mediating roles of inflammatory biomarkers in a cross-sectional study from NHANES 2011-2018. *Sci Rep*. (2025) 15:6617. 10.1038/s41598-025-90131-y 39994278 PMC11850801

[7] Wang B Li L Tang Y Lin T Wu J Wang G et al.. Changes in non-high-density lipoprotein to high-density lipoprotein ratio (NHHR) and cardiovascular disease: insights from CHARLS. *Lipids Health Dis*. (2025) 24:112. 10.1186/s12944-025-02536-3 40133921 PMC11934592

[8] Yu B Li M Yu Z Zheng T Feng X Gao A et al.. The non-high-density lipoprotein cholesterol to high-density lipoprotein cholesterol ratio (NHHR) as a predictor of all-cause and cardiovascular mortality in US adults with diabetes or prediabetes: NHANES 1999-2018. *BMC Med*. (2024) 22:317. 10.1186/s12916-024-03536-3 39113030 PMC11304565

[9] Shan F Ye M Wu H Zhou H Zhong Z Wu Y. Non-HDL/HDL cholesterol ratio (NHHR) as a novel predictor of gestational diabetes mellitus: a NHANES-based cross-sectional study. *J Matern Fetal Neonatal Med*. (2025) 38:2516261. 10.1080/14767058.2025.2516261 40533393

[10] Huang H Yu X Jiang S Wang C Chen Z Chen D et al.. The relationship between serum lipid with sarcopenia: results from the NHANES 2011-2018 and bidirectional Mendelian randomization study. *Exp Gerontol*. (2024) 196:112560. 10.1016/j.exger.2024.112560 39214262

[11] Hua N Qin C Wu F Wang A Chen J Zhang Q. High-density lipoprotein cholesterol level and risk of muscle strength decline and sarcopenia in older adults. *Clin Nutr*. (2024) 43:2289–95. 10.1016/j.clnu.2024.08.017 39217844

[12] Chen LK Woo J Assantachai P Auyeung TW Chou MY Iijima K et al.. Asian working group for sarcopenia: 2019 consensus update on sarcopenia diagnosis and treatment. *J Am Med Dir Assoc.* (2020) 21:300.e–7.e. 10.1016/j.jamda.2019.12.012 32033882

[13] Wang R Wang Y Wei Z Wang J Tang H Gao X et al.. The association between HDL-c levels and computed tomography-based osteosarcopenia in older adults. *BMC Musculoskelet Disord*. (2024) 25:932. 10.1186/s12891-024-08059-9 39563297 PMC11577723

[14] Su X Kong Y Peng D. Evidence for changing lipid management strategy to focus on non-high density lipoprotein cholesterol. *Lipids Health Dis*. (2019) 18:134. 10.1186/s12944-019-1080-x 31170997 PMC6554877

[15] Li B Zha Y Deng M Niu L Li X Zhu R et al.. The association between non-high-density lipoprotein cholesterol to high-density lipoprotein cholesterol ratio and the risk of insulin resistance: results from the NHANES 2003-2016. *BMC Endocr Disord*. (2025) 25:161. 10.1186/s12902-025-01982-5 40597961 PMC12220638

[16] Liu L Liu S Liao Y Zhang X Wang M Lin L et al.. Association of cumulative non-high-density lipoprotein cholesterol to high-density lipoprotein cholesterol ratio with the risk of cardiometabolic disease. *Front Cardiovasc Med*. (2024) 11:1500025. 10.3389/fcvm.2024.1500025 39635266 PMC11614763

[17] Amigó N Torné P Nordestgaard LT Di Giacomo-Barbagallo F Merino C Magni P et al.. Triglycerides as determinants of global lipoprotein derangement: implications for cardiovascular prevention. *Int J Mol Sci*. (2025) 26:8284. 10.3390/ijms26178284 40943205 PMC12427746

[18] Di Giacomo Barbagallo F Bosco G Di Marco M Scilletta S Miano N Martedì M et al.. Assessment of N/L ratio and subclinical atherosclerosis in FH subjects with or without LDLR mutation. *J Endocr Soc.* (2026) 10:bvag001. 10.1210/jendso/bvag001 41700215 PMC12906952

[19] Yanming M Lingjiang L Renji W Xiaojun Y Yinguang W Ruoyu L et al.. Association between non-high-density lipoprotein cholesterol to high-density lipoprotein cholesterol ratio and sarcopenia based on NHANES. *Sci Rep*. (2024) 14:30166. 10.1038/s41598-024-81830-z 39627325 PMC11615221

[20] Yang X Zhong Z. The association between non-high-density lipoprotein cholesterol to high-density lipoprotein cholesterol ratio (NHHR) and sarcopenia: a cross-sectional study. *Exp Gerontol*. (2025) 200:112680. 10.1016/j.exger.2025.112680 39793630

[21] Hao JQ Zhuang ZX Hu SY Zhang YJ Zhang JW He FJ et al.. The association between non-high-density lipoprotein cholesterol to high-density lipoprotein cholesterol ratio (NHHR) and low muscle mass in adults aged 20-59: a population-based study in the United States. *Lipids Health Dis*. (2024) 23:274. 10.1186/s12944-024-02243-5 39198823 PMC11350999

[22] Lin Y Shi X Wu W Chen X. Association of non-high-density lipoprotein cholesterol to high-density lipoprotein cholesterol (NHHR) and sarcopenia in elderly adults. *Front Nutr*. (2025) 12:1614263. 10.3389/fnut.2025.1614263 40661690 PMC12256216

[23] Sun C Jiang H Zhu H Luo Z Wang S. Association between the non-high-density lipoprotein cholesterol to high-density lipoprotein cholesterol ratio and sarcopenia: evidence from CHARLS. *Front Public Health*. (2025) 13:1585986. 10.3389/fpubh.2025.1585986 40371274 PMC12074908

